# Serum metabolomic profiling unveils distinct sex-related metabolic patterns in NAFLD

**DOI:** 10.3389/fendo.2023.1230457

**Published:** 2023-10-03

**Authors:** Charalambos Fotakis, Ioanna-Panagiota Kalafati, Athina I. Amanatidou, Vasiliki Andreou, Manolis Matzapetakis, Maria Kafyra, Iraklis Varlamis, Maria Zervou, George V. Dedoussis

**Affiliations:** ^1^Institute of Chemical Biology, National Hellenic Research Foundation, Athens, Greece; ^2^Department of Nutrition and Dietetics, School of Health Science and Education, Harokopio University of Athens, Athens, Greece; ^3^Department of Informatics and Telematics, Harokopio University of Athens, Athens, Greece

**Keywords:** NMR metabolomics, NAFLD, serum metabolite markers, amino acids, sex, ketone bodies, glycine neurotransmitter, adolescent obesity

## Abstract

**Objective:**

Obesity poses an increased risk for the onset of Nonalcoholic fatty liver disease (NAFLD). The influence of other factors, such as sex in the incidence and severity of this liver disease has not yet been fully elucidated. Thus, we aimed to identify the NAFLD serum metabolic signatures associated with sex in normal, overweight and obese patients and to associate the metabolite fluctuations across the increasing liver steatosis stages.

**Methods and results:**

Using nuclear magnetic resonance (NMR) serum samples of 210 NAFLD cases and control individuals diagnosed with liver U/S, our untargeted metabolomics enquiry provided a sex distinct metabolic bouquet. Increased levels of alanine, histidine and tyrosine are associated with severity of NAFLD in both men and women. Moreover, higher serum concentrations of valine, aspartic acid and mannose were positively associated with the progression of NAFLD among the male subjects, while a negative association was observed with the levels of creatine, phosphorylcholine and acetic acid. On the other hand, glucose was positively associated with the progression of NAFLD among the female subjects, while levels of threonine were negatively related. Fluctuations in ketone bodies acetoacetate and acetone were also observed among the female subjects probing a significant reduction in the circulatory levels of the former in NAFLD cases. A complex glycine response to hepatic steatosis of the female subjects deserves further investigation.

**Conclusion:**

Results of this study aspire to address the paucity of data on sex differences regarding NAFLD pathogenesis. Targeted circulatory metabolome measurements could be used as diagnostic markers for the distinct stages of NAFLD in each sex and eventually aid in the development of novel sex-related therapeutic options.

## 1 Introduction

The liver constitutes an essential organ of the body, performing over 500 vital functions. Its utility may be hampered by the emergence of cardiometabolic disorders, such as obesity, type II diabetes (T2D), dyslipidemia, and insulin resistance (IR). The latter may be the advent of non-alcoholic fatty liver disease (NAFLD), a clinicopathological syndrome closely linked to metabolic stress and genetic predisposition, thus posing a serious threat to human health (1).

NAFLD ranges from hepatic steatosis to non-alcoholic steatohepatitis (NASH), liver fibrosis and liver cirrhosis. In the absence of approved treatments of NAFLD, multiple pharmaceuticals are in different phases of clinical trials targeting mainly bile acid homeostasis, insulin sensitivity improvement, inflammation and lipid metabolism (2). According to Practice Guidance from the American Association for the study of liver diseases NAFLD management should mainly include lifestyle interventions, while pharmacotherapeutics should be administered mainly to patients with biopsy-proven NASH with and without T2D. Drugs may include antihyperglycaemic agents as glucagon-like peptide-1 receptor (GLP-1R) agonists, stimulators of the nuclear receptor peroxisome proliferator-activated receptor-γ (PPAR-γ) and inhibitors of sodium–glucose co-transporter 2 (SGLT-2), while for patients with severe obesity bariatric surgery is recommended (3).

In the era of personalized medicine, there is an increasing concern for the stratification of NAFLD patients according to their distinct phenotype i.e. presence or absence of comorbidities as obesity, T2D, hypertension and hyperlipidemia or even genetic profiling and the need for targeted therapies (4). Moreover, despite the fact that major risk factors towards the development of NAFLD as metabolic syndrome (MetS), visceral adiposity, T2D display sex related differences only a limited research attempts to discern a male-female distinct profile in NAFLD (5, 6). The major findings of the limited population-based studies highlight the higher incidence of NAFLD in men than premenopausal women with the risk of developing NAFLD increasing after menopause (5). NASH-related cirrhosis may advance to hepatocellular carcinoma (HCC) with male patients being four times higher than females probing again the protective role of estrogen until menopause (5).

Besides, sex differences in socio-cultural characteristics, such as dietary trends and exercise should also be considered in NAFLD in addition to the critical influence of age and hormonal status in order to determine disease risk assessment and precise treatment (5).

The implementation of the metabolomics approach may enable monitoring of metabolic perturbations as response to the transition from a healthy and functional liver to lipid overloaded or even inflamed liver. Thus, it may pose a suitable vehicle to uncover the metabolic alterations implicated in the onset or progression of nonalcoholic liver damage.

NMR is a robust analytical high-throughput platform with applications in the search of disease biomarkers (7). Thus, NMR metabolomics can quantitatively frame the variations of metabolites in biological matrices and pinpoint the affected metabolic pathways.

Metabolomics approaches have been applied in a number of clinical studies in order to determine biomarkers associated with NAFLD and NASH as a noninvasive approach informative of the disease status or even prognosis tool (8–11).

The influence of sex in the incidence of liver diseases is rather complex due to the inter-individual heterogeneity. In order to clarify this, we aspired to first identify the NAFLD metabolic signatures associated with each sex, and secondly to discover the association between metabolite fluctuations and the progression of the disease. Therefore, our research contributes to further understand the pathogenesis and the occurring physiological and pathological alterations observed in the presence of NAFLD. This could provide useful information to be employed in the disease early diagnosis tools and treatment improvement.

## 2 Materials and methods

### 2.1 Sample set

Herein, a subsample of 210 NAFLD cases and control individuals from a Greek case-control study was used and detailed methodology can be found elsewhere (12). Adult individuals with no self-declared concomitant liver injury at the time of recruitment were screened for NAFLD. Exclusion criteria included any liver disease, chronic viral hepatitis, hepatotoxic drugs exposure, excessive alcohol consumption, a life-threatening disease or psychiatric disorders impairing the patient’s ability to provide written informed consent, as well as pregnancy or lactation. All study subjects were informed about the aims of the study and signed a written consent. This study was approved by the Ethics Committee of Harokopio University of Athens (38074/13-07-2012), based on the Helsinki Declaration.

#### 2.1.1 NAFLD diagnosis and classification

All volunteers underwent a liver ultrasound (U/S) to determine the stage of NAFLD and to further classify them into four groups based on the result: i)individuals with no hepatic steatosis, ii) individuals with mild (grade 1) hepatic steatosis, iii) individuals with moderate (grade 2) hepatic steatosis and iv) individuals with severe (grade 3) hepatic steatosis. Detailed methodology of disease diagnosis has been mentioned in detail elsewhere (12). Furthermore, due to statistically not different clinical profiles, healthy individuals and those with mild hepatic steatosis (grade 1 subjects) were further classified as controls, whereas individuals with moderate (grade 2 subjects) and severe hepatic steatosis (grade 3 subjects) were classified as cases (12).

#### 2.1.2 Data collection and assessment of sample set characteristics

Participants underwent anthropometric measurements. An interview regarding demographic, family, and individual medical history was also conducted. Blood tests were undertaken after a 12-hour overnight fast and included lipidemic and glycemic profile, as well as liver enzymes. Low-density lipoprotein cholesterol (LDL-C) was calculated using the Friedewald equation and the degree of insulin resistance was determined by the homeostatic model assessment (HOMA-IR) (13, 14). Fatty Liver Index (FLI) includes Body Mass Index (BMI), waist circumference (WC), triglycerides (TG) and gamma-GT and has achieved an accuracy of 0.84 in detecting fatty liver (15).

Quantitative variables are described as mean ± SD and categorical variables as relative frequencies. Pearson chi-square test was applied to test independence between categorical variables while one-way ANOVA was used to test differences among the different disease stages. Pairwise comparisons were performed using the Bonferroni correction (**Table 1**).

**Table 1:** Characteristics of the sample set.

	No hepatic steatosis (n=39)	Grade I (n=119)	Grade 2 (n=43)	Grade 3 (n=9)	p
Age (years)	38.08 ± 11.54**^†,^**^¶,§^	45.91 ± 10.89**^†^**	47.72 ± 10.62^¶^	49.89 ± 11.1^§^	<0.001
Gender (females - %)	46.2	58.0	62.8	77.8	0.254
BMI (kg/m^2^)	23.76 ± 3.41**^†,^**^¶^	25.32 ± 3.54^*,#^	30.3 ± 5.28^†,*^	32.67 ± 5.43^¶,#^	<0.001
WHR	0.79 ± 0.07**^†,^**^¶^	0.84 ± 0.1^*,#^	0.89 ± 0.08^†,*^	0.94 ± 0.1^¶,#^	<0.001
AST (U/L)	21.13 ± 5.07	20.82 ± 6.22	21.23 ± 6.02	23.56 ± 5.96	0.617
ALT (U/L)	21 ± 9.53**^†^**	21.73 ± 11.38^¶^	24.70 ± 9.62	35.11 ± 19.08**^†,^**^¶^	0.003
gammaGT (U/L)	18.95 ± 20.26	18.64 ± 15.79	22.81 ± 18.70	35.11 ± 29.28	0.045
FGlu (mg/dL)	82.03 ± 7.26**^†,^**^¶^	83.67 ± 7.28^*,#^	89.12 ± 10.91^†,^^*^	96.56 ± 18.25^¶,#^	<0.001
FIns(μU/mL)	9.36 ± 3.48**^†,^**^¶^	9.92 ± 5.38^*,#^	12.94 ± 6.68^†,^^*,**^	20.15 ± 14.92^¶,#,**^	<0.001
HOMA-IR	1.96 ± 0.79**^†,^**^¶^	2.07 ± 1.20^*,#^	2.84 ± 1.42^†,^^*,**^	5.08 ± 4.69^¶,#,**^	<0.001
TC (mg/dL)	177.18 ± 36.69**^†,^**^¶,*^	197.24 ± 35.24**^†^**	204.63 ± 35^¶^	219 ± 34.57^*^	0.001
LDL (mg/dL)	105.44 ± 30.67**^†,^**^¶,*^	122.62 ± 32.68**^†^**	127.66 ± 30.96^¶^	133.83 ± 27.11^*^	0.005
HDL (mg/dL)	59.03 ± 13.24	57.58 ± 13.92	57.66 ± 13.47	58.17 ± 11.32	0.950
TG (mg/dL)	63.56 ± 24.98**^†,^**^¶,*^	82.50 ± 39.30**^†,^**^#^	96.79 ± 36.41^¶,**^	136.33 ± 65.65^*,#,**^	<0.001
FLI	13.99 ± 16.19^†,¶^	23.23 ± 21.06^*,#^	50.75 ± 27.55^†,*,**^	75.76 ± 15.37^¶,#,**^	2.5201E-19
Hyperlipidemia (%)	15.4	42.9	44.2	66.7	0.004
DMII (%)	5.1	0	11.6	33.3	<0.001
Hypertension (%)	20.5	22.7	46.5	77.8	<0.001
MetS (%)	5.1	9.2	14	44.4	0.006

Values given as mean ± SD for quantitative variables and relative frequencies (%) for categorical variables. p-value: One-way ANOVA p -value for quantitative and Pearson chi-square p-value for categorical variables. BMI, Body mass index; WHR, Waist-to-hip ratio; AST, Aspartate transaminase; ALT, Alanine transaminase; gammaGT, Gamma-glutamyltransferase; FGlu, Fasting glucose; FIns, Fasting insulin; HOMA-IR, Homeostasis Model Assessment – Insulin Resistance; TC, Total cholesterol; LDL, Low-density lipoprotein; HDL, High-density lipoprotein; TG, Triglycerides; FLI, Fatty Liver Index; DMII; Diabetes Mellitus II; MetS, Metabolic Syndrome.. **†,**¶,§,*,#,**: p ≤ 0.05 for multiple comparisons using the Bonferroni method.

### 2.2 Serum samples collection and pretreatment

Serum samples collected from the individuals were thawed at room temperature and extracted according to Nagana Gowda et al. (16) protocol. In particular, 250 μL serum samples were extracted with 500 μL methanol (1:2 v/v), vortexed and placed at -20 °C for 20 min. Samples were centrifuged (11.000 rpm/4 °C) for 20 min to allow protein parts to precipitate. Supernatants were collected and evaporated to dryness. Samples were reconstituted to 400 μL D_2_O and 150 μL phosphate buffer (0.2M, Na_2_HPO_4_ 2H_2_O and NaH_2_PO_4_, pH=7.0) with internal standard trimethylsilyl propionic acid sodium salt (TSP) (2.75 mM) and were transferred to 5 mm NMR tubes for ^1^H-NMR analysis.

### 2.3 ^1^H-NMR spectroscopy

^1^H-NMR spectra were acquired using a Varian 600 MHz NMR spectrometer equipped with a 1H{13C/15N} 5mm PFG Automatable Triple Resonance probe at room temperature (25 °C). The CPMG pulse sequence with presaturation for water suppression was applied. In total, 128 transients were collected with 64 K data points, a relaxation delay of 5 s and an acquisition time of 4 s, Receiver gain was kept constant for all acquisitions. ^1^H NMR spectra were referenced at TSP chemical shift (0.00 ppm) and processed with 0.3 exponential line broadening (17).

### 2.4 Data handling and metabolite assignment

^1^H-NMR spectra were preprocessed with MestreNova (v. 10.1, Santiago de Compostela, Spain) software. Manual phase correction, automatic baseline correction and sinc apodization were applied to improve spectra resolution. Binning of 0.001 ppm was selected. The water D_2_O region (4.68 - 5.00 ppm) was excluded together with the peak at 3.36 ppm attributed to methanol residual. The spectra were normalized to the standardized area of the reference compound (TSP), manually aligned and converted into ASCII format using the software Mnova ver.10.1.

#### 2.4.1 Annotation of metabolites

Metaboneer, an in-house fully automated metabolite identification platform (18), facilitated the resonance-peak identification of 42 metabolites in serum (**Table S1**). The identification procedure was also assisted by literature data as well as by 2D NMR experiments (gCOSY, zTOCSY, gHMBCad and gHSQCad), their acquisition parameters are described in **Table S2**. **Table S3** presents the resonance peaks assignments of the identified metabolites.

### 2.5 Statistical analysis

#### 2.5.1 Postprocessing of spectral data

SIMCA-P (version 14.0, Umetrics, Umeå, Sweden) was applied. The spectral data were mean-centered and Pareto (Par) scaled. The application of Par scaling allows any metabolites of low-medium intensity to affect the analysis only if they represent systematic variation. The unsupervised Principal Component Analysis (PCA), as well as the supervised Orthogonal Projections to Latent Structures Discriminant Analysis (OPLS-DA) (19) multivariate models were extracted at a confidence level of 95%.

First, the exploratory PCA was applied to the mean-centered and scaled data in order to acquire a general insight and visualize any relation (trends, outliers) among the observations (samples). A PCA model estimates the systematic variation in a data matrix by a low dimensional model plane. Then, classification analysis ensued by further subjecting the data set to OPLS-DA, an extension of the supervised partial least square (PLS) regression method. This increases the quality of the classification model by separating the systematic variation in X into two parts, one that is linearly related to Y (predictive information), and one that is unrelated to Y (orthogonal information). The predictive information of Y in X is concentrated in the first predictive component and is associated with the between groups variation while the variation in X which is unrelated to Y is put in the second and orthogonal component and is linked to the within groups variation. The cognition of the orthogonal variation improves model visualization and interpretation. The concept of these methods has been extensively discussed (20).

#### 2.5.2 Identification of important features

S-line plots were extracted to pinpoint the metabolites that contributed to the samples’ discrimination. These plots utilize color coding visualization in order to detect the metabolites that influence most the group membership and explain the between samples’ variation.

#### 2.5.3 Model validation

The quality of models (PCA/OPLS-DA) was described by the goodness-of-fit R^2^ (0≤R^2^ ≤1) and the predictive ability Q^2^ (0≤Q^2^ ≤1) values that were extracted according to the internal cross-validation default method of the software SIMCA-P. The goodness-of-fit, R^2^ explains the variation in the model, thus constituting a quantitative measure of how well the data of the training set was mathematically reproduced. Particularly, the OPLS-DA models reliability was assessed by the cumulative Q^2^ which is considered a de facto default diagnostic parameter for validating OPLS-DA models in metabolomics. It represents the fraction of the variation of Y that could be predicted by the model and the goodness of prediction issuing values greater or equal to 0.4. The difference between R^2^Y(cum) and the cumulative Q^2^ remained always lower than 0.3 (R^2^- Q^2^>0.3), indicative of avoidance of overfitting the data. The statistical significance of the regression models was tested through the cross-validation analysis of variance (CV-ANOVA) diagnostic issuing *p-*value<0.05. Furthermore, the performance of the regression models was validated through permutation tests using 999 random permutations of the Y matrix and evaluating the response curves of R^2^Y and Q^2^Y of the permutated models versus the real model to evaluate whether the specific classification of two classes in a model were significantly better than any other models obtained by randomly permuting the original group’s attribution (21). An additional measure of PLS-DA model validity included the extraction of receiver-operating characteristic (ROC) curves to assess the ability of the PLS latent variable Tpred to correctly classify the test set. Moreover, by evaluating the area under receiver-operating characteristic curve (AUROC) statistics, we assessed the diagnostic accuracy of a marker as low, fair and superior when the area under the ROC (AUROC) curve reached values of 0.5 < AUC < 0.7, 0.7 < AUC < 0.9 and AUC > 0.9, respectively (17).

#### 2.5.4 Metabolic markers and associated metabolic pathways

The web-based MetaboAnalyst (V5.0) platform (https://www.metaboanalyst.ca/) was utilized for biomarker discovery, classification and pathway mapping of metabolites exhibiting AUROCs>0.7 to enable exploration of disease-related metabolites and pinpoint the most relevant pathways.

The ASCII file containing the aligned spectra after their reduction into spectral buckets of 0.001 ppm was used as the input data type for analysis with MetaboAnalyst. From the available modules we also facilitated Biomarker Analysis to extract ROC curves.

The levels of metabolites exhibiting sub-optimal and higher performance for each control and NAFLD samples of male and female sex have been framed in box plots after performing adjustments for the confounding factor BMI, to account for population heterogeneity.

The metabolites exhibiting fair and superior AUROC accuracy (AUROC>0.7) compiled a one-column compound list that was in turn implemented for Enrichment Analysis and Pathway Analysis.

Specifically, the metabolic pathway analysis (MetPA) algorithms included hypergeometric test for over-representation analysis and Relative-betweenness centrality for pathway topology analysis while the KEGG Homo sapiens library was applied. Metabolic pathways with hypergeometric test p-value less than 0.05 were considered to be disturbed. Metabolite Set Enrichment Analysis (MSEA), was also performed for the metabolites exhibiting AUROC > 0.7 incorporating to the aforementioned one-column compound list the corresponding fold changes. MSEA monitors whether these metabolites are represented more often than expected by chance and in an attempt to identify biologically meaningful patterns.

Furthermore, the derived disease metabolic markers were used as a query to the network explorer module (Network Analysis) of MetaboAnalyst in order to uncover potential meaningful links with disease phenotypes. To generate a Metabolite-Disease Interaction Network, the input metabolites are mapped to the selected interaction network to create subnetworks containing these seeds and their direct neighbors (i.e. first-order subnetworks).

The associations were obtained from HMDB. A degree cutoff:2 and betweenness cutoff: 1, were selected. In particular, for the samples of male sex a subnetwork with 6 nodes, 7 edges and 4 seeds was generated, while for the samples of female sex a subnetwork with 8 nodes, 14 edges and 4 seeds was generated.

## 3 Results

The sample set of this secondary analysis included 210 individuals, among which 39 were NAFLD-free, 119 had mild hepatic steatosis, 43 moderate and 9 had severe NAFLD. Approximately 61% of the sample consisted of females. It was observed that the greater the disease severity the higher the age of the individuals (p<0.001). Anthropometric indices (i.e., BMI and WHR) were higher among the higher disease stage groups (p<0.001). Moreover, all biochemical indices significantly increased along with the increase of NAFLD grade, except for AST and HDL levels which were not statistically significant among the different groups. The prevalence of hyperlipidemia, diabetes mellitus II (DMII), hypertension and metabolic syndrome (MetS) were incrementally increased along with the increase of NAFLD severity (p<0.05). Last but not least, FLI was significantly and incrementally increased from patients with no NAFLD to patients with severe NAFLD. Overall, distinct clinical profiles were observed based on NAFLD severity (**Table 1**).

### 3.1 NMR-metabolomics endeavor

The circulatory metabolome provides a rational compartment to interpret metabolite variations, therefore the first step in our endeavor was to implement PCA on the host of serum blood samples.

**Figure 1:**
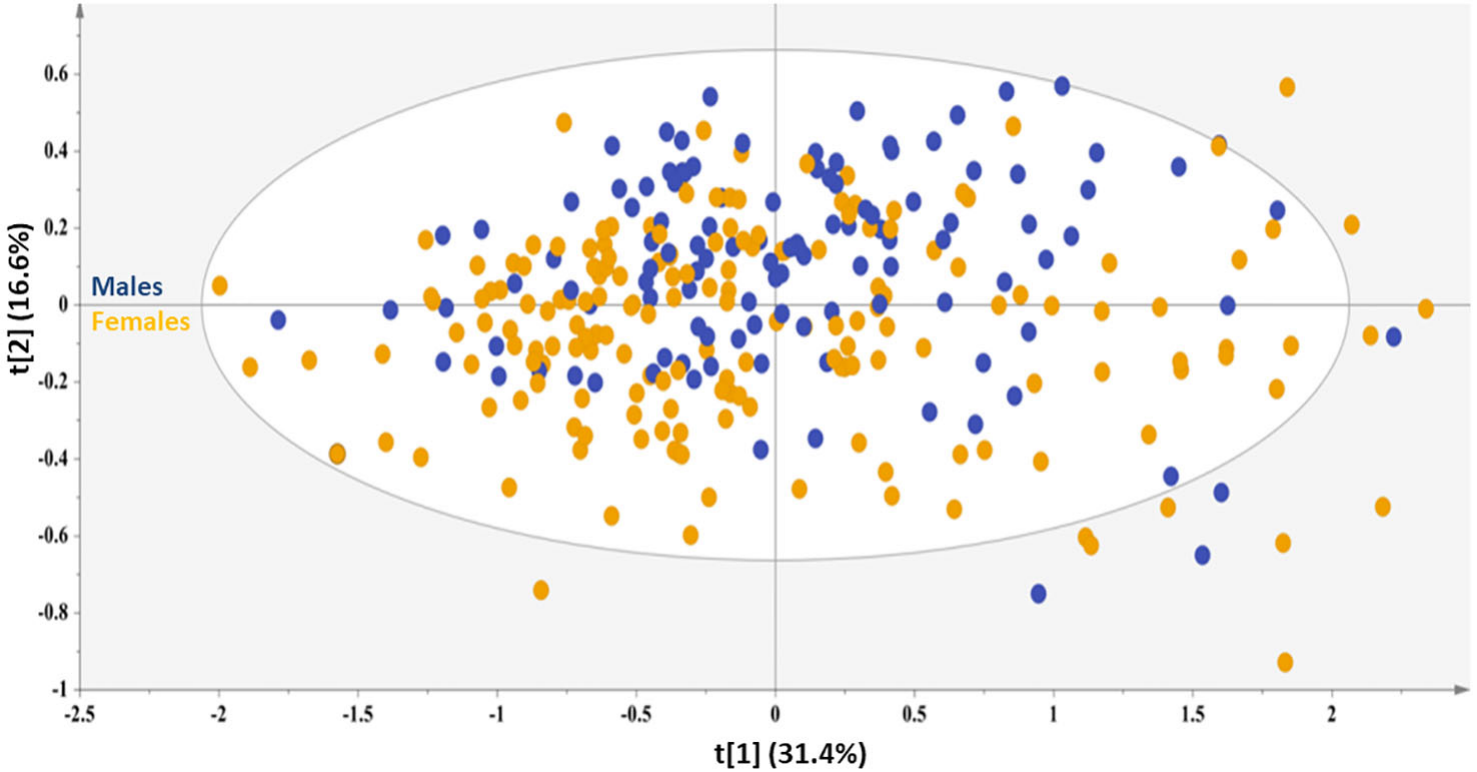
PCA model generated for the total sample set, A=2, N=210; R^2^X(cum)= 0.48, and Q^2^(cum)=0.46. Blue circles =male sex, orange circles=female sex.

In this direction, a PCA model with two components was computed explaining 48.0% of the data variation, featuring good predictability (Q^2^(cum) = 0.46) to provide an overview of the samples, highlighting a possible clustering and pinpointing strong outliers (**Figure 1**). Interestingly, of the several characteristics included in this PCA model, such as the absence or presence of NAFLD pathology of different grades and the presence of comorbidities, such as hyperlipidemia, MetS, DMII and hypertension, the observed trend along the second principal component probed to a differentiation based only on the sex of the subjects. In fact, the samples of male sex mainly localize in the first and second quadrants, while the samples of female sex assemble to a great extend in the third and fourth quadrants. This unsupervised overview highlights sex as a prominent factor for these samples’ differentiation, since the second principal component explains a high amount of variance with an eigenvalue (i.e., 10.5). This result further enhances the notion that liver is metabolically distinct for each sex (6), herein literature has noted that hepatic injury and inflammation are related to sex and diverse sex hormone levels. Therefore, in an attempt to alleviate the impact of this factor, each sex was further explored under the scope of OPLS-DA models.

#### 3.1.1 NAFLD discriminating metabolites of the male subjects

**Figure 2:**
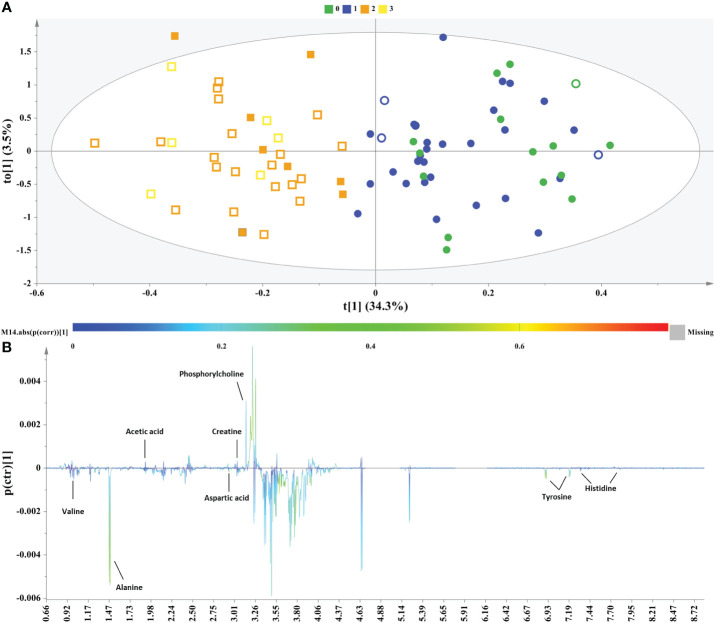
Samples of male sex. **(A)** OPLS−DA model; A=1 + 1, N=80; R^2^X(cum)= 0.38, R^2^Y(cum)=0.75 and Q^2^(cum)=0.46, P-value= 1.29208e-009. Circles= Controls (Healthy & Grade 1 NAFLD), Squares = NAFLD cases (Grade 2 & Grade 3), empty symbols =subjects with MetS. Samples are colored according to NAFLD level: in green (grade 0: healthy); in blue (Grade 1: mild steatosis); in orange (Grade 2: moderate steatosis); and in yellow (Grade 3: severe steatosis), **(B)** S−line plot highlights important features (annotated metabolites) identified by OPLS-DA for the between samples’ variation.

NMR data from the male subjects were subjected to OPLS-DA analysis comparing controls and NAFLD cases to further interrogate the data and elucidate potential markers. The OPLS-DA model clearly resolved the metabolic variation and discriminated two clusters (controls & NAFLD cases) along the first component (**Figure 2A**). The control group only contained healthy liver and grade 1 subjects, while the NAFLD group only included grade 2 and 3 subjects.

Permutation testing with receiver operator characteristic (ROC) curves validated the OPLS-DA discrimination (**Supplementary Figure S1**).

The derived S-line plot framed the between samples variation and highlighted glucogenic amino acids alanine, valine, aspartic acid, histidine and tyrosine, the latter constituting both a glucogenic and ketogenic amino acid as the key metabolites exhibiting a strong correlation with the more advanced stages of NAFLD (grade 2 & 3). On the other hand, the control samples exhibited elevated levels of the creatine, phosphorylcholine and acetic acid (**Figure 2B**).

**Figure 3:**
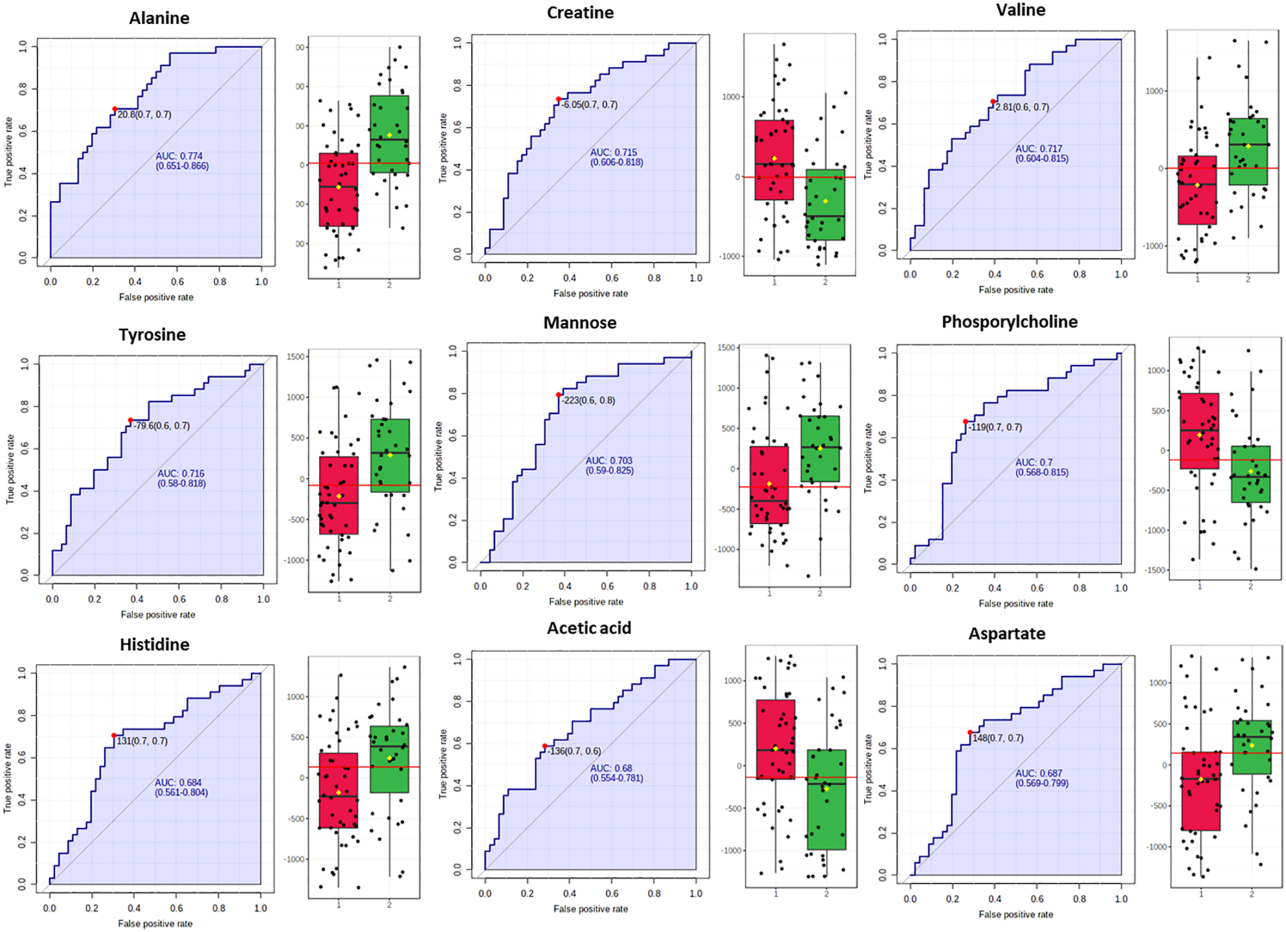
Box plots and ROC curves for each metabolite differentially abundant between controls (in red color) and NAFLD cases (in green color) in male population are depicted. All box plots were extracted after normalization with the BMI.

Biomarker analysis through the MetaboAnalyst platform issued the ROC curves for the differentially abundant metabolites and the areas under curve (AUROC) assessed their discriminatory potential relatively to the impact of NAFLD in serum (**Figure 3**).

In fact, for the male samples no metabolite exhibited AUROC > 0.9, while alanine, valine, mannose, tyrosine, phospshorylcholine and creatine exhibited AUROC > 0.7 probing them as the most fitting metabolite markers of NAFLD in men. The metabolites histidine, acetic acid and aspartic acid displayed sub-optimal AUROC, slightly less than 0.7 (**Figure 3**).

#### 3.1.2 NAFLD discriminating metabolites of the female subjects

**Figure 4:**
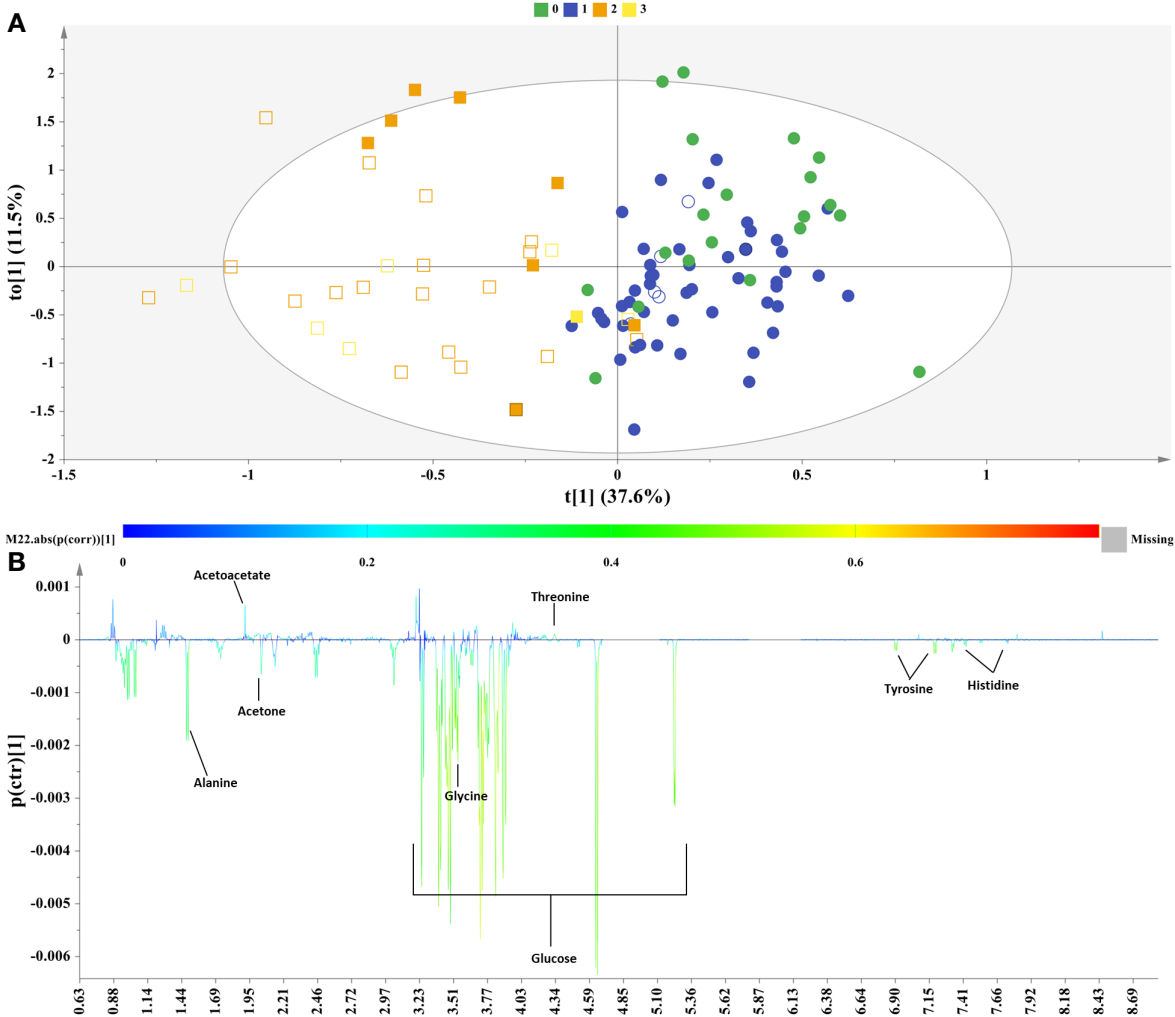
Samples of female sex. **(A)** OPLS−DA model; A=1 + 1, N=107; R^2^X(cum)= 0.49, R^2^Y(cum)=0.63 and Q^2^(cum)=0.4, P-value= 1.11316e-012. Circles= Controls (Healthy & Grade 1 NAFLD), Squares = NAFLD cases (Grade 2 & Grade 3), empty symbols =samples with MetS. Samples are colored according to NAFLD level: in green (grade 0: healthy); in blue (Grade 1: mild steatosis); in orange (Grade 2: moderate steatosis); and in yellow (Grade 3: severe steatosis), **(B)** S−line plot highlights important features (annotated metabolites) identified by OPLS-DA for the between samples’ variation.

Subsequently, the class information in relation to the control or NAFLD state of each female sample was incorporated into an OPLS-DA model to elicit potential discriminative biomarkers. **Figure 4A** illustrates the OPLS-DA scores and in **Figure 4B** the S-line plot of the studied groups. As also observed for the male sample set, the extracted OPLS-DA model regarding the female samples clearly discriminated along the first component the controls (healthy & grade 1) from NAFLD cases (grade 2 & 3). The S-line plot highlighted increased levels of the glucogenic amino acids histidine, alanine, and tyrosine the latter being also a ketogenic amino acid, as well as glucose, glycine and the ketone body acetone associated with the NAFLD cases. On the other hand, the control samples display higher levels of threonine and acetoacetate (**Figure 4B**).

The OPLS-DA models have been validated through permutation testing and receiver operator characteristic (ROC) curves (**Supplementary Figure S2**).

**Figure 5:**
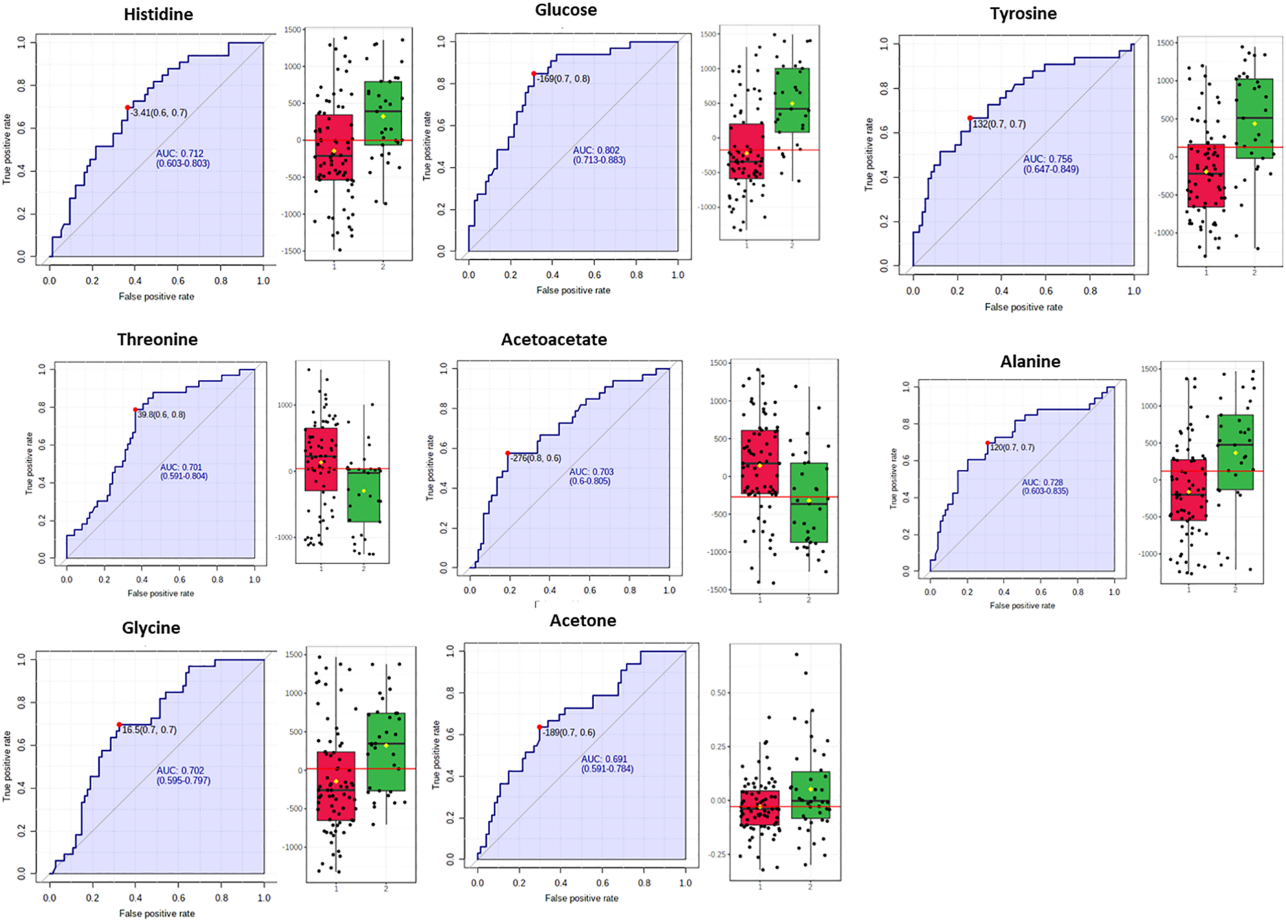
Box plots and ROC curves for each metabolite differentially abundant between controls in red color and NAFLD cases (in green color) in female population are depicted. All box plots were extracted after normalization with the BMI.

The S-line plot (**Figure 4B**) revealed that the metabolic profile of females diagnosed with NAFLD bears similarities to that of the male sex incorporating similarly mainly both glucogenic and ketogenic amino acids, but it also exhibits distinct metabolic signatures. Biomarker analysis and ROC statistics evaluated further the discriminatory potential of the OPLS-DA derived metabolites. Thus, for the samples of female sex no metabolite exhibited AUROC > 0.9, though histidine, tyrosine, alanine, glucose, glycine, threonine and acetoacetate exhibited AUROC > 0.7 qualifying them as the most fitting metabolite markers of NAFLD in women. The metabolite acetone emerged with sub-optimal AUROC slightly less than 0.7 (**Figure 5**).

### 3.2 Metabolic pathways involved in NAFLD

**Figure 6:**
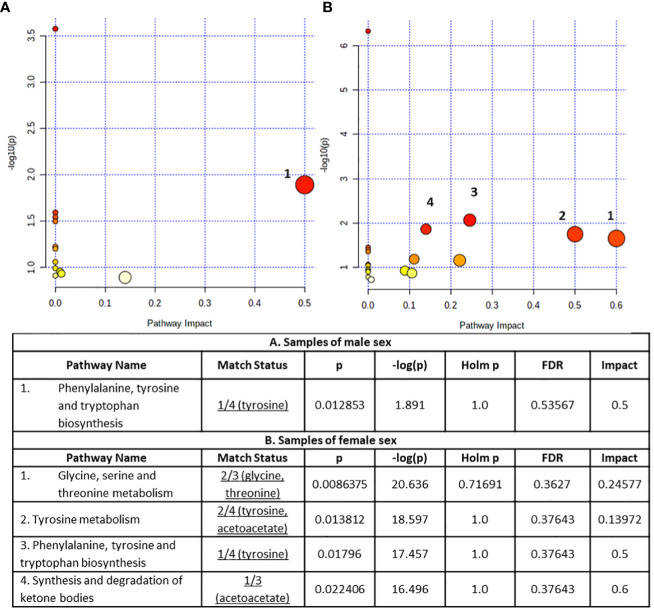
Summary plots for metabolic pathway analysis, **(A)** for samples of male sex **(B)** for samples of female sex. Every circle represents one pathway, and the deeper color represents the more significant changes of the metabolites in the related pathway based on P value. The size of the circle varies accordingly to the higher centrality of the metabolite in the related pathways (impact value).

The altered metabolites exhibiting AUROC>0.7 were submitted to the Pathway analysis module of MetaboAnalyst 5.0 to identify perturbed metabolic pathways in response to NAFLD (**Figure 6**).

The obtained results concerning the male sex serum substrate revealed the Phenylalanine, tyrosine and tryptophan biosynthesis pathway as statistically significant (*p* < 0.05) disturbed (**Figure 6A**). On the other hand, the statistically significant perturbed pathways (*p* < 0.05) for the samples of female sex samples included glycine, serine and threonine metabolism, tyrosine metabolism, phenylalanine, tyrosine and tryptophan biosynthesis and synthesis and degradation of ketone bodies with the former two pathways containing at least two metabolites (**Figure 6B**).

**Figure 7:**
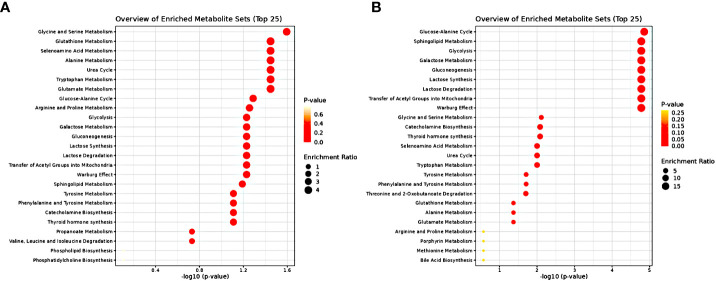
Metabolite Set Enrichment Analysis results **(A)** for samples of male sex **(B)** for samples of female sex. The dot plots present disturbed KEGG metabolic pathways related to significantly differential metabolites. Dot color varies according to the significance level of the identified pathways (P value) and dot size is calculated on the basis of the enrichment ratio (observed hits/expected hits).

In order to further assess the underlying biological perturbation we also implemented Metabolite Set Enrichment Analysis (**Figures 7A, B**). In fact, for the male samples the altered metabolites were mainly involved in the Glycine and Serine Metabolism, Glutathione Metabolism, Selenoamino Acid Metabolism, Alanine Metabolism, Urea Cycle, Tryptophan Metabolism and Glutamate Metabolism. Whereas for the female samples the altered metabolites were mainly involved in the Glucose-Alanine Cycle, Sphingolipid Metabolism, Glycolysis, Galactose Metabolism, Gluconeogenesis, Lactose Synthesis, Lactose Degradation, Transfer of Acetyl Groups into Mitochondria, Warburg Effect, Glycine and Serine Metabolism, Catecholamine Biosynthesis, Thyroid hormone synthesis, Selenoamino Acid Metabolism, Urea Cycle, Tryptophan Metabolism, Tyrosine Metabolism, Phenylalanine and Tyrosine Metabolism, Threonine and 2-Oxobutanoate Degradation, Glutathione Metabolism, Alanine Metabolism and Glutamate Metabolism.

### 3.3 Metabolite –disease relation network analysis

The revealed metabolites exhibiting AUROC>0.7 were uploaded to the Network Explorer analysis module of Metaboanalyst platform and a Metabolite-Disease Interaction Network was extracted identifying metabolite-disease connections that cross pathway boundaries. The exploration of disease-related metabolites for male and female sex samples is displayed in (**Supplementary Figures S4A, B**) respectively. Briefly, a link between liver steatosis and mental health was highlighted in both sexes.

## 4 Discussion

An insulin-resistant state, being a common driver in the development and progression of NAFLD, is associated with dysregulated glucose metabolism in the liver, muscle and adipocytes and increased lipolysis in the latter followed by free fatty acids release upregulation (8). IR and lipotoxicity resulting from increased delivery of free fatty acids or *de novo* lipogenesis within the liver induces mitochondrial dysfunction and oxidative stress due to overloaded lipid β-oxidation (22). IR in muscle cells leads to excess protein catabolism and increased released levels of amino acids. In fact, patients with NAFLD usually exhibit alterations in circulating amino acids, including increases in branched chain amino acids and aromatic amino acids and decrease in amino acids related to glutathione (GSH) metabolism as potential impact to increased demand for GSH synthesis in response to oxidative stress (8).

Accumulating evidence supports the sexually dimorphic nature of NAFLD/NASH by means of significant differences in the pathophysiology, epidemiology and clinical outcomes between men and women (5, 23, 24). Clinical observational studies confirm the higher prevalence of non-alcoholic fatty liver in men until the age of 50-60 years, while afterwards the NAFLD incidence becomes similar between the two sexes as a result of the women menopause state (24). Moreover, NAFLD/NASH severity is more pronounced in male individuals than females (23). Although few studies distinguish pre- versus post-menopausal women, evidence suggest that women at reproductive age are protected against fatty liver disease through estrogen signaling mainly via Estrogen receptors alpha (ERα) to improve liver insulin sensitivity and limit hepatic fat accumulation (25–27). Animal studies probe to a differentiated hepatic metabolism pattern of dietary amino acids due to the effect of estrogen (25). Premenopausal women display higher insulin sensitivity than men at the hole-body level resulting in better glucose homeostasis and are at lower risk of developing NAFLD and T2D (24). Moreover, healthy premenopausal women are more protective from cardiovascular disease (CVD) as compared with men (28). The onset of menopause features proatherogenic circulating metabolome alterations mirroring female sex hormones fluctuations and predisposing women to atherosclerotic and cardiovascular disease. Estrogen exposure may partially modify this metabolomic shift (29).

Biomarker and pathway analyses probed clearly to an imbalanced metabolic network associated with the pathogenesis of NAFLD in both sexes and more importantly probed to sex-dependent distinct metabolic signatures.

### 4.1 Identified common NAFLD metabolic signatures between male and female sex

Our study revealed higher serum concentration of the glucogenic and ketogenic amino acid tyrosine and of the glucogenic amino acids alanine and histidine, in both sexes with NAFLD (grade 2 & 3) and fluctuations of ketone bodies or acetic acid. In fact, NAFLD severity was positively correlated with the serum levels of tyrosine (AUROC=0.716 for male and AUROC=0.756 for female), alanine (AUROC=0.774 for male and AUROC=0.728 for female) and histidine (AUROC=0.684 for male and AUROC=0.712 for female) (**Figures 3**, **5**).

Fluctuations in circulating aromatic amino acids are often detected in individuals with NAFLD. High tyrosine concentration in patients with NAFLD has been highlighted in a number of publications (8) possibly indicating a dysregulation in hepatic metabolism of this amino acid (30). Furthermore, increased levels of tyrosine has been observed related to IR (30, 31) and varying proportional to the severity of the fibrotic stage (31). Besides, phenylalanine is irreversibly converted to tyrosine mainly in the liver (30), thus the aromatic amino acid biosynthetic pathway emerges disturbed in both sexes (**Figure 6**).

Increased circulating alanine levels in NAFLD patients have also been linked with hepatic IR (8, 30), which is associated with the pathogenesis of NAFLD. Through the glucose–alanine cycle, alanine’s release from the muscle during starvation and delivery in the liver enables its conversion to pyruvic acid, which enters in the gluconeogenic pathway and regenerates glucose. Under this prism, the revealed imbalance in the glucose–alanine cycle and alanine metabolism (**Figure 7**) probes to a disturbed liver-skeletal muscle metabolic crosstalk (32). Recent findings point to both transcriptionally and non-transcriptionally regulation of amino acid metabolism by glucagon. In this line, elevated concentrations of alanine have been pinpointed in subjects with NAFLD and hyperglucagonemia (33).

The implemented biomarker analysis highlighted the significant increase of histidine along the progression of NAFLD for the female subjects and a respective increase for the male subjects. Few studies to date underline the contribution of histidine to the NAFLD pathogenesis (34). Of note, an animal study probed to the contribution of a histidine-excess diet in rats to the high occurrence of minor fibrosis and liver cell damage (35).

### 4.2 Identified male distinct NAFLD metabolic signatures

Higher serum concentrations of valine, aspartic acid and mannose were positively associated with the progression of NAFLD among the male subjects while a negative association was observed with the levels of creatine, phosphorylcholine and acetic acid (**Figure 3**).

Valine displayed significant increase (AUROC=0.717) in male NAFLD subjects. A sufficient body of evidence supports the positive correlation of high circulatory BCAAs with liver diseases including NAFLD occurrence (36). It has been observed that males with NAFLD have greater plasma levels of valine, leucine, and isoleucine than females while a sex-dependent relationship of plasma BCAAs with NAFLD severity has been reported (37). Animal studies have probed the expression of estrogen receptor ERa in the liver as key regulator of hepatic lipid metabolism highlighting also a differentiated metabolic function between the two sexes. Interestingly, the BCAA metabolism emerges as the most affected pathway due to the lack of hepatic ERa particularly in males (38).

Mannose has been also revealed as significantly increased in NAFLD male subjects (AUROC=0.703). Mannose is related to hepatic glycogen breakdown and has been associated together with Valine, Isoleucine and Glutamate among other metabolites with increased risk of developing T2D in a large prospective study (39).

Aspartic acid has emerged bearing sub-optimal statistical significance (AUROC=0.687). Interestingly, aspartic acid is involved in urea cycle, an essential hepatic metabolic function which is compromised in NAFLD and this may potentially link to the increased blood concentration of involved amino acids (40). Urea cycle dysregulation induces increased ammonia levels in liver and blood and could be the driving cause for hepatic inflammatory response and fibrosis, cognitive dysfunction, secondary sarcopenia, immune dysfunction and malignancy (41).

Creatine, phosphorylcholine and acetic acid were elucidated as metabolites related to a healthier liver, which are consistent with previous researches. Particularly, creatine which can be obtained from dietary sources but it is also *de novo* synthesized in the liver, has been observed in elevated concentration in the healthy male subjects (AUROC=0.715). Other *in vivo* and *in vitro* researches have portrayed that treatment with creatine, aids in the prevention of causes for fatty liver disease. In fact, the expression of transcription factors (PPARα and PPARγ) and of key genes related to fatty acid metabolism is modulated by creatine (42). Thus, PPAR agonists are intensively investigated in the treatment of NAFLD (43).

Furthermore, the healthy male subjects displayed higher levels of phosphorylcholine (AUROC=0.7), a precursor and a breakdown product of phosphatidylcholine. Low levels of phosphatidylcholine, which is vital for cell membranes and lipoproteins, have been shown to enhance fat accumulation and drive *de novo* lipogenesis (44). Hepatic phosphatidylcholine metabolism is linked to NAFLD (45), and variations in phospholipids subclasses have been documented in subjects with and without NASH. Interestingly, NAFLD/NASH patients have been found with a lower phosphatidylcholine-to-phosphatidylethanolamine ratio than healthy patients (46).

Acetic acid was also pinpointed as a potential marker for male healthy liver although with sub-optimal significance (AUROC=0.68). This metabolite deriving from the diet or gut microbiota fermentation has an important influence on fatty acid metabolism and may suppress NAFLD/NASH development by regulating hepatic lipid metabolism and insulin sensitivity via its receptor FFAR2 (free fatty acid receptor 2) in hepatocytes (47). In this direction, animal studies have highlighted acetic acid as an important metabolite in the hindrance of lipid accumulation as it advances lipolysis as well as fatty acid oxidation and inhibits fatty acid synthesis (48).

### 4.3 Identified female distinct NAFLD metabolic signatures

The metabolites glucose, glycine, acetoacetate, acetone and threonine were distinctly associated with the progression of NAFLD among the female subjects (**Figure 5**).

In the liver, insulin fails to stimulate glucose metabolism when in an insulin-resistant (IR) state and, as a result the production of endogenous glucose increases and insufficient glucose disposal occurs. Congruent with this supposition our study highlighted the increased concentration of serum glucose in NAFLD female subjects as compared to controls (AUROC=0.802). Previously, when the isotope tracer approach was applied, it pinpointed that NAFLD patients produced elevated glucose concentration (hepatic insulin resistance) and lower glucose uptake (peripheral insulin resistance) in spite of increased insulin levels in comparison to the healthy subjects (49). Estrogens acting mainly through ERα receptors suppress hepatic glucose production by reducing gluconeogenesis and increasing glycogen synthesis and storage (25). The higher circulating glucose level in Grade 2 & 3 NAFLD female subjects possibly mirrors the lack of estrogens due to menopause status as can be inferred by the age characteristics of the female subjects, (48.63 ± 10.84) years for moderate and (53.43 ± 6.80) for severe hepatic steatosis (**Table S4**). In accordance, MSEA revealed the disturbed Glycolysis and Gluconeogenesis pathways (**Figure 7B**).

Glycine serum concentration was revealed as significantly disturbed between controls and NAFLD cases (AUROC=0.702) (**Figure 5**). Besides its dietary source, glycine is also biosynthesized mainly from serine derived from diet or *de novo* synthesized from the 3P-glycerate intermediate of the glycolysis pathway mainly in the kidneys. Through one-carbon metabolism glycine is involved in the biosynthesis of purines and of the major intracellular antioxidant glutathione (50). In accordance, metabolite pathway analysis and MSEA revealed the disturbed Glycine and Serine Metabolism and the Glutathione metabolism (**Figures 6B**, **7B**). The pathogenic role of oxidative stress on NAFLD is well established and decreased levels of glycine are reported in NAFLD patients (50). Furthermore, transcriptomics analysis in the livers of NASH patients have revealed suppression of glycine biosynthetic genes (51). Thus, the apparent increase of glycine concentration in NAFLD cases as revealed from our study is inconsistent with literature. This triggered our attention to explore further this important neurotransmitter’s variation through the different stages of hepatic steatosis. Analysis of variance (ANOVA) (p= 0.049076) through box–plots presentation (**Supplementary Figure S3**) revealed the trend in each hepatic steatosis stage. An initial decrease of glycine serum concentration in mild hepatic steatosis was followed by a recovery in moderate hepatic steatosis probably attributed to a transient response to oxidative stress or associated to microbial metabolism following a special dietary approach while the severe hepatic steatosis clearly featured a significant decay of glycine abundance in accordance with literature (**Supplementary Figure S3**). Perturbations in the levels of glycine have been associated to menopausal hormone therapy, albeit this warrants further investigation (29).

Our study elucidated fluctuations in the concentrations of ketone bodies, acetoacetate (AUROC=0.703) and acetone (AUROC=0.691), (**Figure 5**), the former bearing higher statistical significance. It has been postulated that ketogenesis holds a critical role in hepatic lipid disposal and is activated in states of increased fatty acids, decreased carbohydrates and/or low circulating insulin (52). Increased hepatic influx of fatty acids upregulates the mitochondrial β-oxidation pathway and the release of acetyl-CoA which can be directed towards ketogenesis or enter the TCA cycle and either catabolized to citrate and exported to the cytoplasm directed towards *de-novo* lipogenesis or oxidized and induce gluconeogenesis. Impairment in ketogenesis diverts acetyl-CoA in increased rates of gluconeogenesis and *de-novo* lipogenesis thus promoting NAFLD pathogenesis (52, 53). Evidence from human and animal studies supports the hypothesis that ketogenesis is initially increased in NAFLD in response to the increased liver lipid accumulation, followed by an impaired mechanism as NAFLD progresses reflecting in the reduction in circulatory ketone body levels (53). In a recent large prospective Dutch cohort study, circulating ketone bodies were upregulated in subjects suspected with NAFLD as determined from the calculated fatty liver index (FLI), while NAFLD and higher circulating ketone bodies were related to a high risk of all‐cause mortality (54). On the other hand, using stable isotope tracers, a study demonstrated that NAFLD subjects (24-hour-fasted) displayed resistance to ketosis associated with increased disposal of acetyl-CoA in the TCA cycle and increased hepatic glucose production (55).

Our study prompts also to an impaired ketogenic pathway mirrored in the significantly decreased levels of acetoacetate associated with the moderate and severe hepatic steatosis. Moreover, the higher circulating glucose level observed also in those subjects potentially advocate to a concomitant divergence of acetyl-CoA in the TCA cycle as an adaptive mechanism in agreement with literature. The complex interplay of the disturbed metabolic pathways is revealed in the metabolite pathway analysis and the MSEA (**Figures 6B**, **7B**) including synthesis and degradation of ketone bodies and gluconeogenesis, glycolysis which generates pyruvate which either oxidates to acetyl-CoA (transfer of Acetyl Groups into Mitochondria) or converts to lactate (Warburg effect) which via inhibition of Histone Deacetylase (HDAC) stimulates *de-novo* lipogenesis pathway (56).

Interventions and therapeutics targeting ketogenic pathway activation holds promise for the treatment of NAFLD. Peroxisome proliferator-activated receptor α (PPARα) is a ligand activated transcription factor which mediates the expression of a number of genes involved in hepatic lipid metabolism including ketogenesis (57). Selective PPARa modulators are extensively investigated for NAFLD/NASH management (58).

Finally, we noted higher serum levels of threonine in healthy women as compared with NAFLD subjects (AUROC=0.701), (**Figure 5**). MSEA has prompt to a disturbed Threonine and 2-Oxobutanoate Degradation pathway among NAFLD female subjects (**Figure 7B**). It is reported that supplements of threonine for mice in a high-fat diet alleviated the onset of fat deposition (59). Of note, a community-based case-control study including senior Chinese aged over 65 years has showed that threonine, valine and lysine were negatively correlated with NAFLD risk and highlighted the potential beneficial effect of the increased consumption of food rich in those essential amino acids as eggs, milk and deep-sea fish (60).

### 4.4 Impact of NAFLD on disease occurrence

The metabolic markers were used as a query in the Metabolite-Disease Interaction Network, in an effort to uncover potential associations of NAFLD with other medical conditions (**Supplementary Figures S4A, B**).

The obtained results in male subjects point towards a link between NAFLF and risk for lung cancer. This finding is consistent with recent research evidence stating that obesity and NAFLD might consist a risk factor for lung adenocarcinoma although the underlying causality is under investigation (61, 62). Furthermore, a large cohort prospective study in China demonstrated that men with NAFLD have a higher risk of developing all cancers in particular thyroid and lung cancer and revealed also a positive association between the latter and smoking (63).

The results from the Metabolite-Disease Interaction Network also indicated a potential relation between NAFLD and schizophrenia in both men and women. Interestingly, this link was established based on different metabolites sets in each sex. NAFLD/NASH is highly comorbid with mental illness and research efforts attempt to establish the underlying linking mechanisms at genetic, epigenetic, metabolic and inflammatory level, even though the exposure to antipsychotic drugs and unhealthy lifestyle behavior (64–66). In our study, only two participants were under antidepressant medication according to their statement thus antipsychotic drugs influence is unlikely. Perturbation of common metabolic pathways interrelating schizophrenia and NAFLD merits further investigation through longitudinal studies. A recent study overcame the potential bias due to the metabolic side effects of antipsychotic drugs by addressing first-episode psychosis (FEP) with the inclusion of drug-naïve patients. The study revealed the greater risk of FEP patients to present at least one altered MetS component as compared to control subjects (67).

Moreover, on females with NAFLD the metabolites alterations were linked with the occurrence of Alzheimer’s disease (AD). There is increasing evidence that NAFLD could be associated with moderate cerebral dysfunction and cognitive decline but again more longitudinal studies are needed to confirm if this association is linked to comorbidities as CVD and T2D or hepatic dysfunction and induced neuroinflammation and neurodegeneration (68–70).

Finally, disorders related to severe damage to the liver as tyrosinemia type I (71, 72) and pyridoxamine 5-phosphate oxidase (PNPO) deficiency (73–75), were also suggested for the female subjects with NAFLD. These uncommon metabolic disorders affect a different age range from our sample pool but their emergence in part explains why NAFLD is often associated with aspects of MetS.

The potential link of liver diseases with other medical conditions seems to be supported by multiple studies and should be considered in clinical practice.

## 5 Strengths and limitations

A strength of the present study is that it revealed sex related molecular signatures of fatty liver disease staging in the circulating metabolome, supporting further the sexually dimorphic nature of the disease (5, 23, 24). Within the concept of Precision Medicine integration of OMICS data is mandatory towards tools capable for early diagnosis, for discrimination between low-risk and high-risk profiles and towards the development of effective drugs or treatments targeting well-defined patients subgroups (76, 77). Metabolites, which are different between men and women in NAFLD patients, would represent ideal candidates for a stratified NAFLD risk model.

A limitation of this study is the lack of data on the menopause state for female subjects or even the regularity of the participants’ menstrual cycles, i.e. menstrual diaries or sex hormones measurements. This limitation was discovered in the course of our study which was a secondary analysis of blood sera collected for investigating dietary patterns with the odds for NAFLD and data related to the sex hormones were out of scope then. This lack of data impeded the stratification of women by pre- or post-menopause and did not allow a clear investigation of the hormonal status influence in the metabolite levels. The current literature supports a protective role of estrogens in women and to a lesser degree in men against hepatic steatosis (25, 27). The impact of estrogen deficiency is reflected in the increased risk of NAFLD/NASH in women after menopause, in women following ovariectomy, and in women under antiestrogenic therapy for the treatment of hormone-sensitive breast cancer (27, 78). Of note, long or irregular menstrual cycles are associated with an increased risk of NAFLD (79). Interestingly, studies have associated earlier menarche with NAFLD later in life (25, 43). On the other hand, androgens can act as anti-steatotic agents but as evidenced by recent literature display sex-specific opposing effects. Excess of androgens (hyperandrogenism) is associated with enhanced risk of NAFLD in women as occurs in poly-cystic ovary syndrome while androgenic deficiency (hypogonadism) is associated with an increased risk of NAFLD in men (24, 78). In light of all the above, it becomes evident that consideration of hormonal status may further support causal associations between metabolite alterations and the influence of metabolic dysfunction associated fatty liver disease.

Finally, we would like to stress that there is a need to validate biomarkers identified in this study in additional cohorts if these results are to be considered for clinical use.

## 6 Conclusions

NAFLD prevalence is still growing and its heterogenous landscape encompasses various determinants at genetic and epigenetic level, hormonal, endocrine and metabolic status, environmental exposure, as well as coexistence of comorbidities as obesity, T2D and CVD. Drawing on this, the integration of high-throughput multi-OMICS data could contribute to accurately stratify patients based on their genetic and phenotypic background, to establish diagnostic tools capable of discriminating between low-risk and high-risk profiles and to apply personalized treatment. Metabolome profiling being closely linked to phenotype could play a major role towards the qualification and integration of validated biomarkers in clinical practice for the early noninvasive diagnosis.

Our untargeted NMR metabolomics study addressed the paucity of data on sex differences and revealed a distinct metabolic bouquet due to substantial inter-sex variation in NAFLD progression. Acquired results show that circulatory metabolome profiling could be used as the basis to elicit surrogate markers and generate diagnostic modalities for the distinct stages of NAFLD in each sex. We aspire to further validate the discrimination patterns in larger and diverse patient cohorts. Importantly, the acquired results reflect the even more pressing need of including sex factor, hormonal and menstrual cycle related data in clinical studies related to metabolic dysfunction associated fatty liver disease. Adequate consideration of the substantial inter-sex variation in NAFLD progression and liver-related outcomes is crucial to targeting diagnostic strategies at “at risk” individuals with the burden of NAFLD but also for monitoring the response to treatments.

## Data availability statement

The raw data supporting the conclusions of this article will be made available by the authors, without undue reservation.

## Ethics statement

The studies involving humans were approved by Ethics Committee of Harokopio University of Athens (38074/13-07-2012), based on the Helsinki Declaration. The studies were conducted in accordance with the local legislation and institutional requirements. The participants provided their written informed consent to participate in this study.

## Author contributions

CF: Conceptualization, Methodology, Validation, Visualization, Investigation, Writing- Original draft preparation, I-PK: Conceptualization, Data curation, Investigation, Writing- Original draft preparation. AA: Conceptualization, Data curation, Writing- Original draft preparation, VA: methodology, Data collection and curation, Writing- Original draft preparation, MM: Data collection and curation, Writing-Reviewing & Editing, MK: Data curation, Writing- Original draft preparation, IV: Conceptualization, Data curation, Writing- Original draft preparation, MZ: Conceptualization, Supervision, Writing- Reviewing and Editing, GD: Conceptualization, Supervision, Project administration, Funding acquisition, Writing- Reviewing and Editing. All authors contributed to the article and approved the submitted version.
