# Supplementary material for: Serum metabolomic profiling unveils distinct sex-related metabolic patterns in NAFLD
Source: Front Endocrinol (Lausanne). 2023 Oct 3;14:1230457. doi: 10.3389/fendo.2023.1230457 (PMC10579908; doi:10.3389/fendo.2023.1230457)

Figure S1 Validation of the OPLS-DA model for samples of male gender, A. ROC Curves – AUC(2)=0.764189, AUC(1)=0.764189, B. Permutation testing with 999 permutations

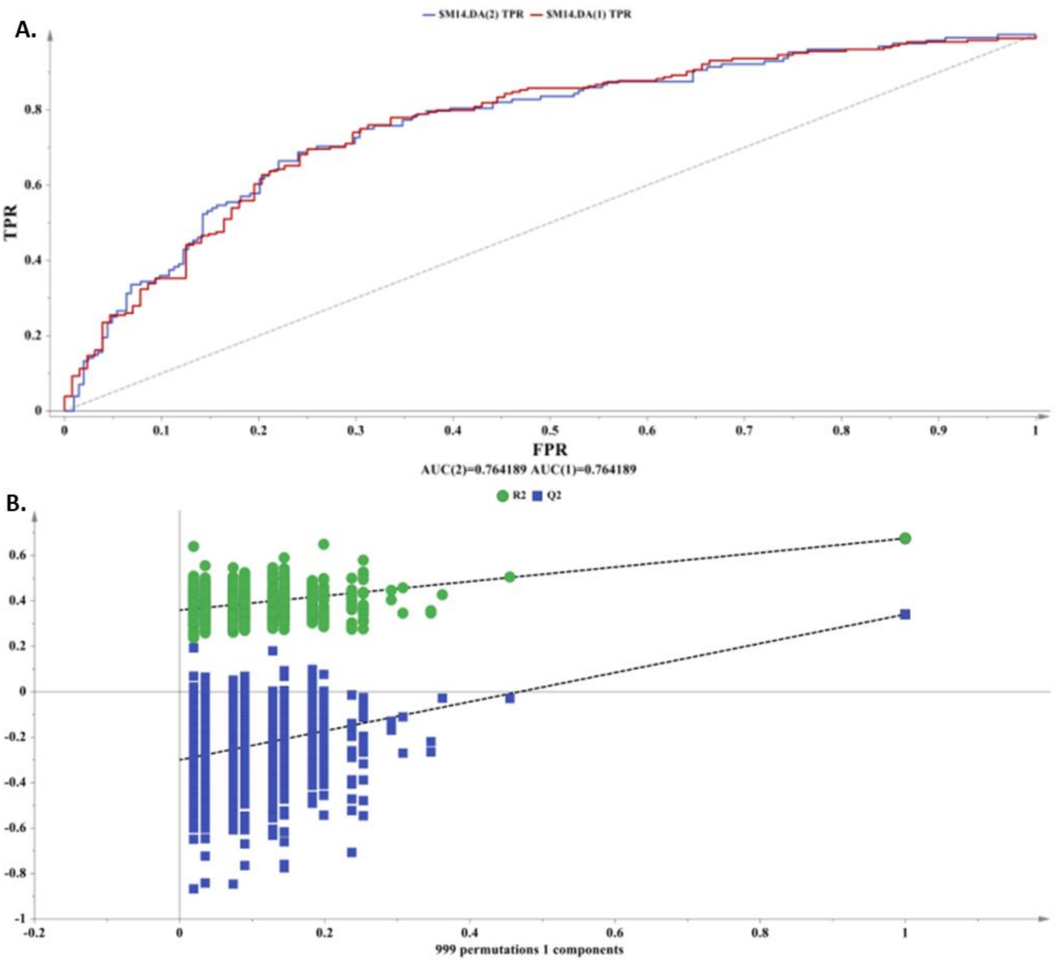

Figure S2 Validation of the OPLS-DA model for samples of female gender A. ROC Curves – AUC(2)=0.718654, AUC(1)=0. 718654, B. Permutation testing with 999 permutations

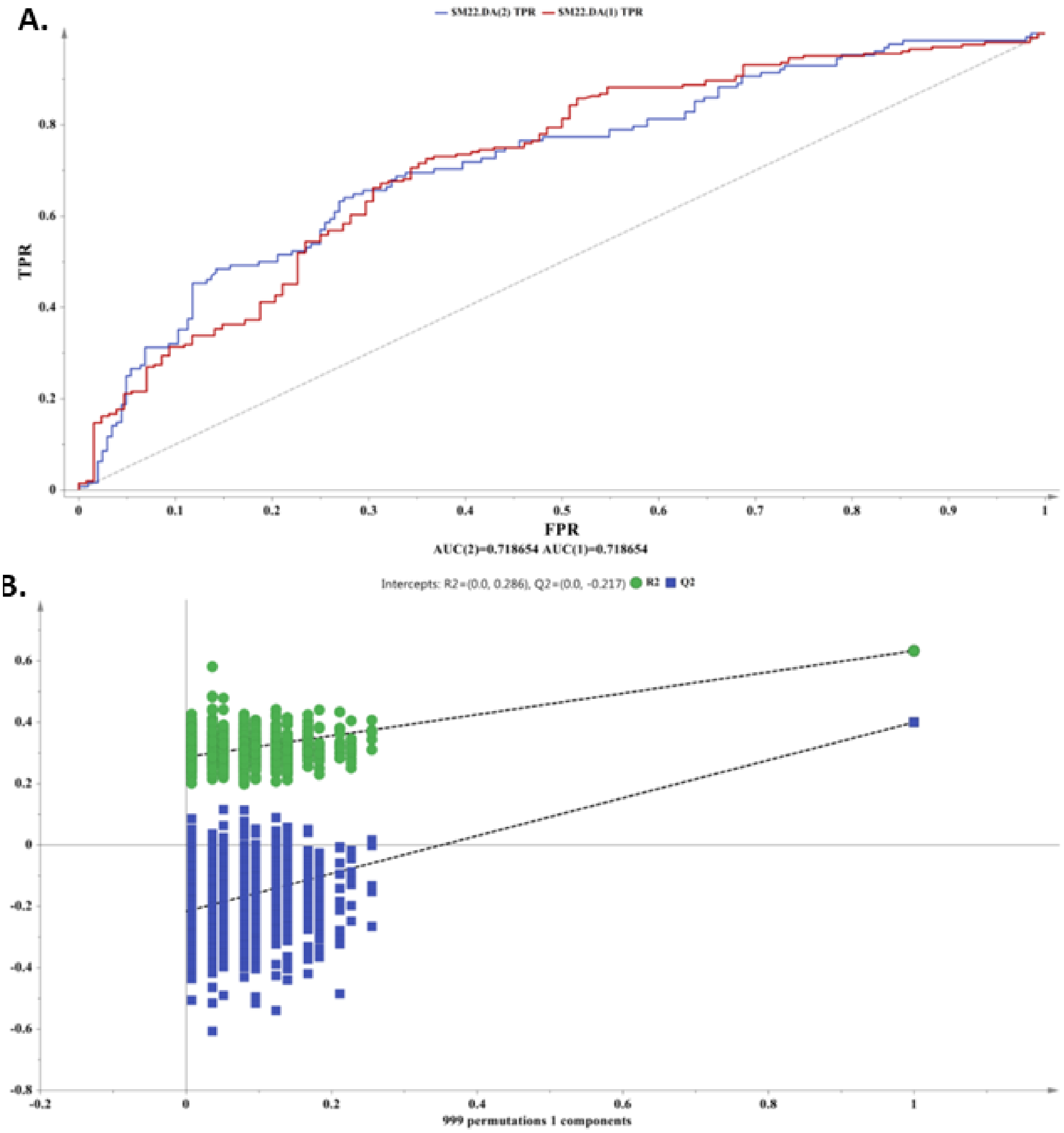

Figure S3 Analysis of Variance (ANOVA) through Box plot presentation exhibits the trend of glycine concentration for samples of female sex in each hepatic steatosis stage.

A decrease in glycine concentration when comparing samples with no hepatic steatosis (grade 0) to those with mild hepatic steatosis (grade 1); A moderate recovery in glycine concentration when comparing samples with no hepatic steatosis (grade 0) to those with hepatic steatosis (grade 2); A significant decay in glycine concentration when comparing samples with no hepatic steatosis (grade 0) to those with severe hepatic steatosis (grade 3).

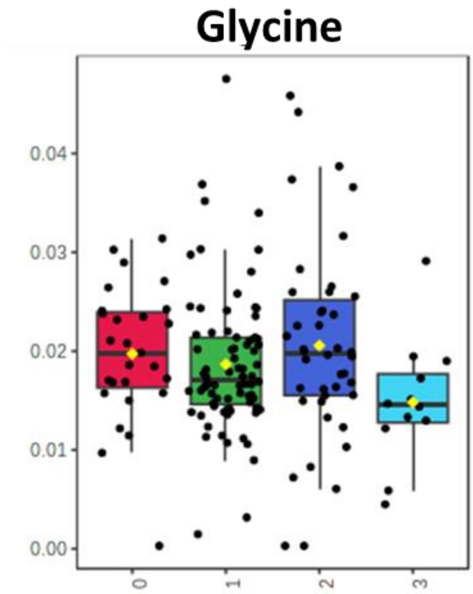

Metabolite	chi.squared	p-value	LOG10(p)	FDR
Glycine	78.563	0.049076	1.3091	0.049076

Figure S4. Schematic diagram illustrating metabolite-disease interaction network based on the NMR revealed differential metabolites and associations retrieved from HMDB from (**A**) male sex samples and from (**B**) female sex samples.

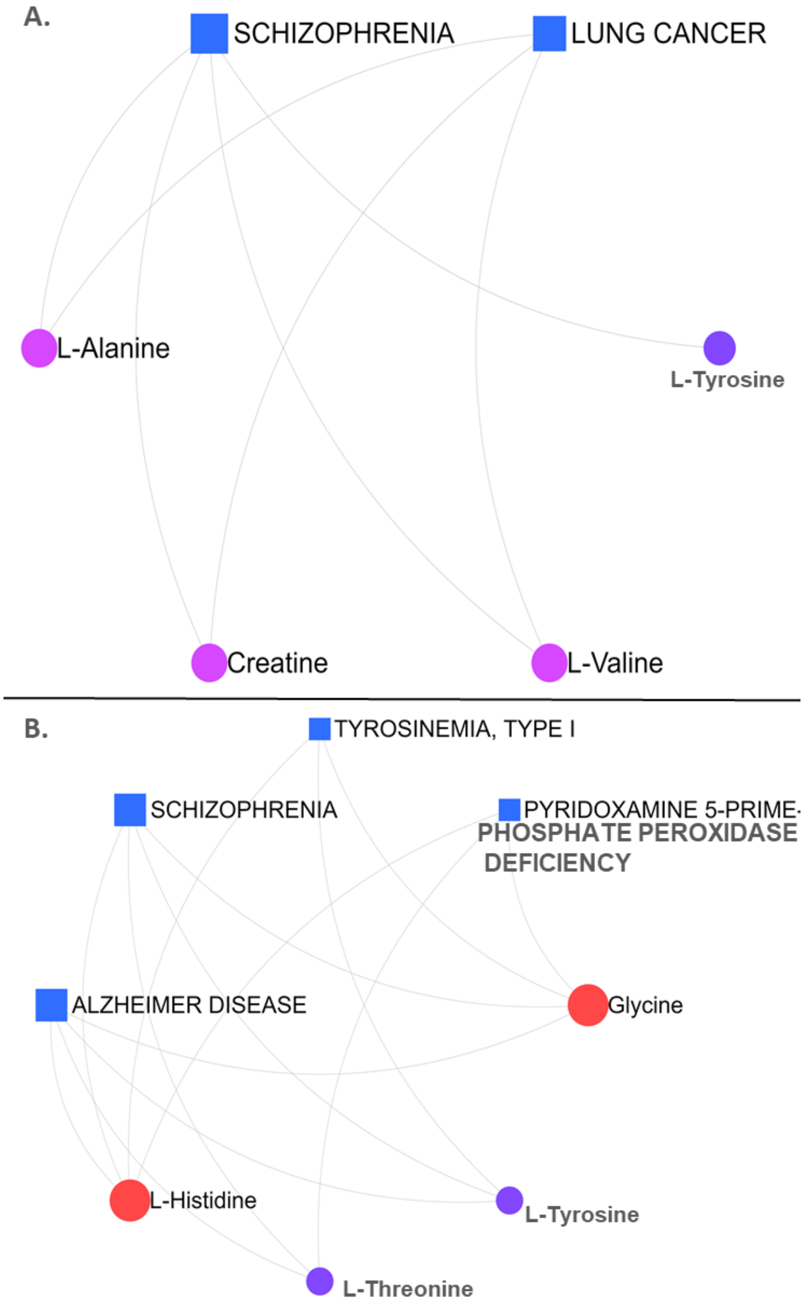

Supplement: Supplementary file 2 [file DataSheet_2.pdf]
